# Supplementary figures and images for: Primate phylogenomics uncovers multiple rapid radiations and ancient interspecific introgression
Source: PLoS Biol. 2020 Dec 3;18(12):e3000954. doi: 10.1371/journal.pbio.3000954 (PMC7738166; doi:10.1371/journal.pbio.3000954)

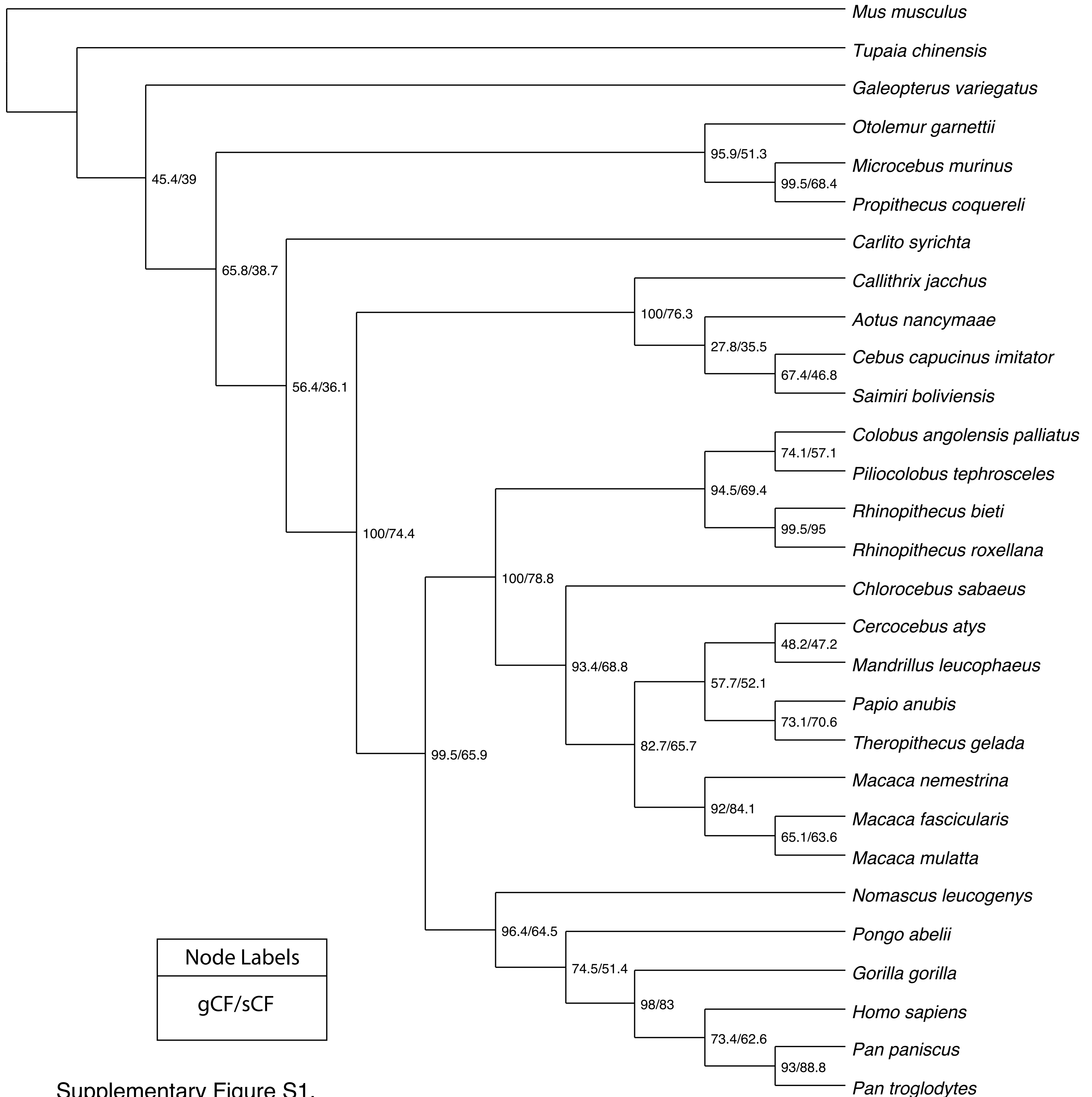

Supplementary Figure S1.

Supplement: S1 Fig — In general, gCFs increase, while the sCFs remain the same, indicating that gene tree error is a likely source of some discordance. gCF, gene concordance factor; sCF, site concordance factor. (PDF) [file pbio.3000954.s001.pdf]

A.

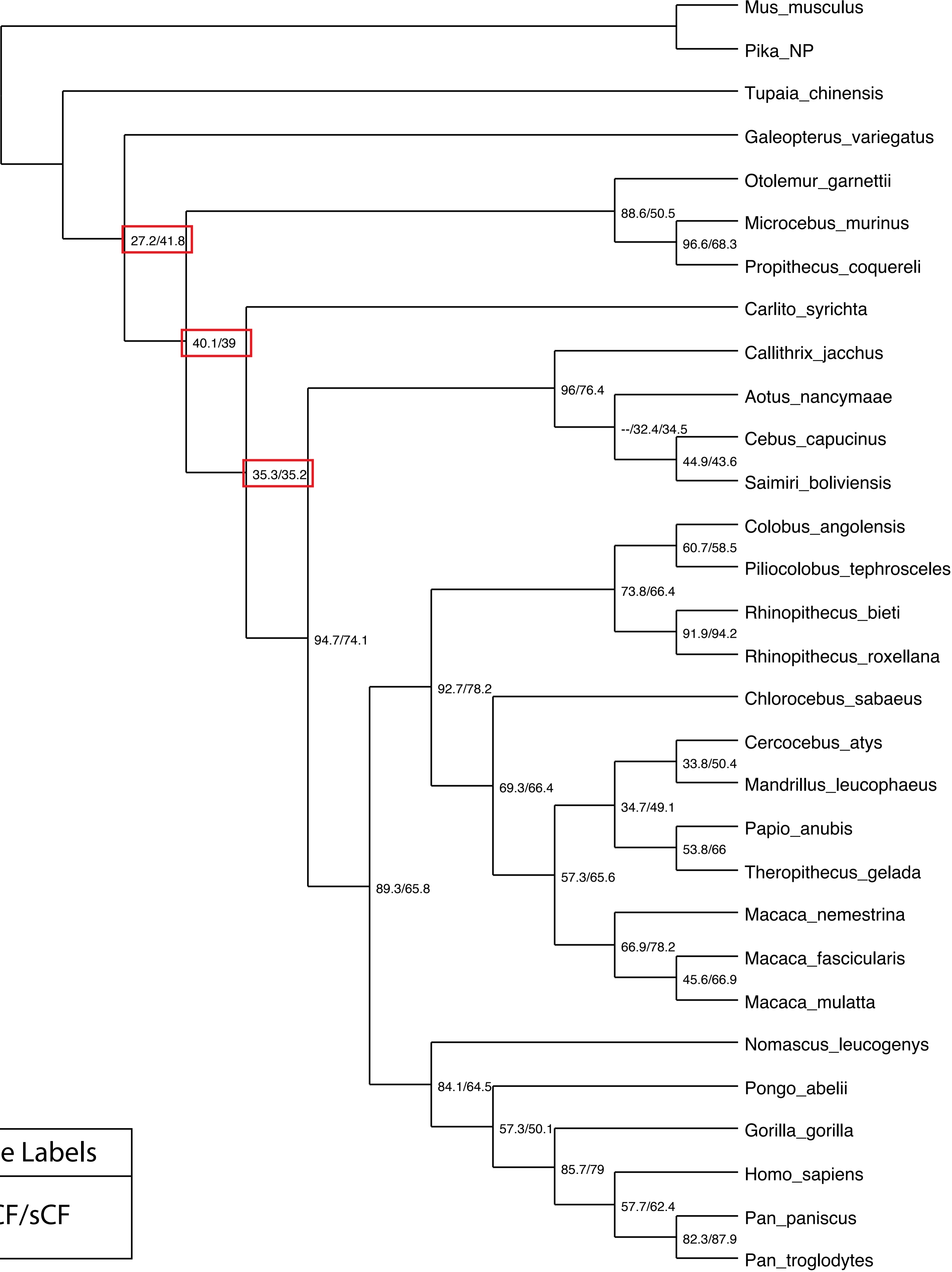

B.

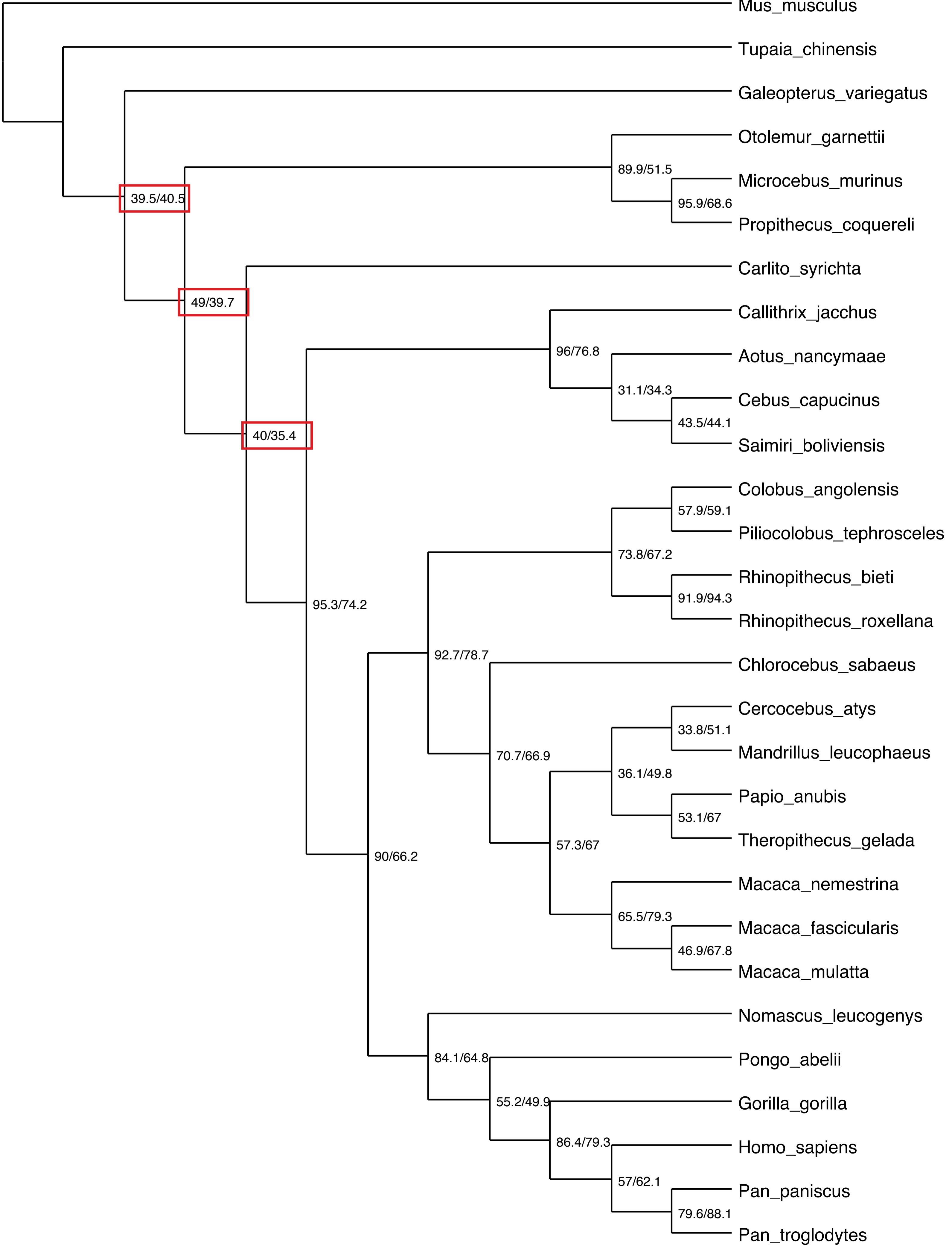

Supplementary Figure S2.

Supplement: S2 Fig — (A) gCFs and sCFs for these 150 genes when pika is included. (B) gCFs and sCFs for these same genes when pika is not included. We observe slightly higher gCFs near the base of the tree with pika excluded (red boxes). Note that these species trees use unit-length branch lengths for readability of branch labels. gCF, gene concordance factor; sCF, site concordance factor. (PDF) [file pbio.3000954.s002.pdf]

# Concordance Factors vs. Node Depth

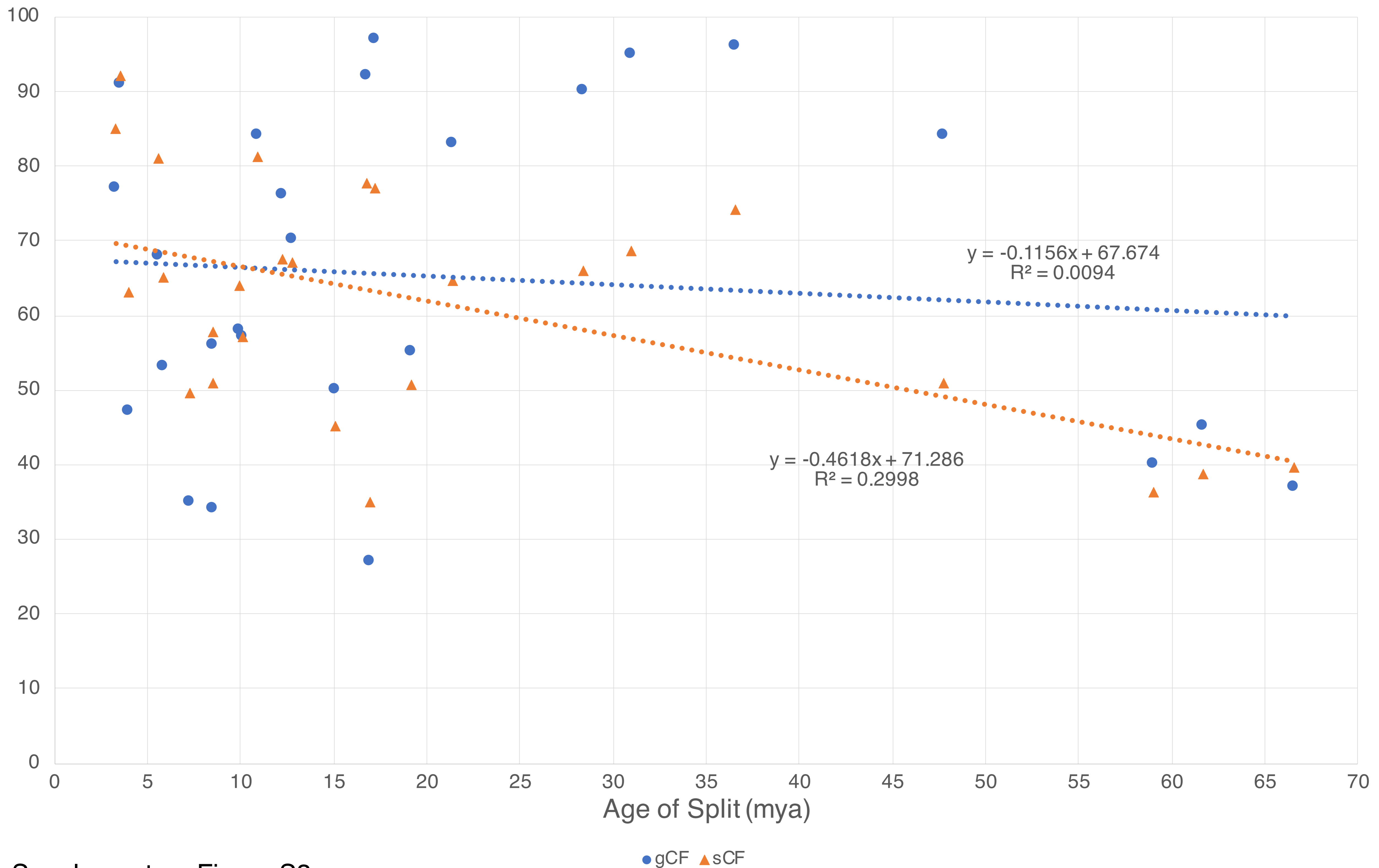

Supplement: S3 Fig — No correlation was found between gCFs and node depth, whereas a slightly negative correlation was found between sCFs and node depth. This relationship indicates that homoplasy may act to slightly reduce sCFs deeper in the tree. The data underlying mean node ages are provided in S1 Data. gCF, gene concordance factor; sCF, site concordance factor. (PDF) [file pbio.3000954.s003.pdf]

A) Per locus mutation rate =  $3 \times 10^{-7}$

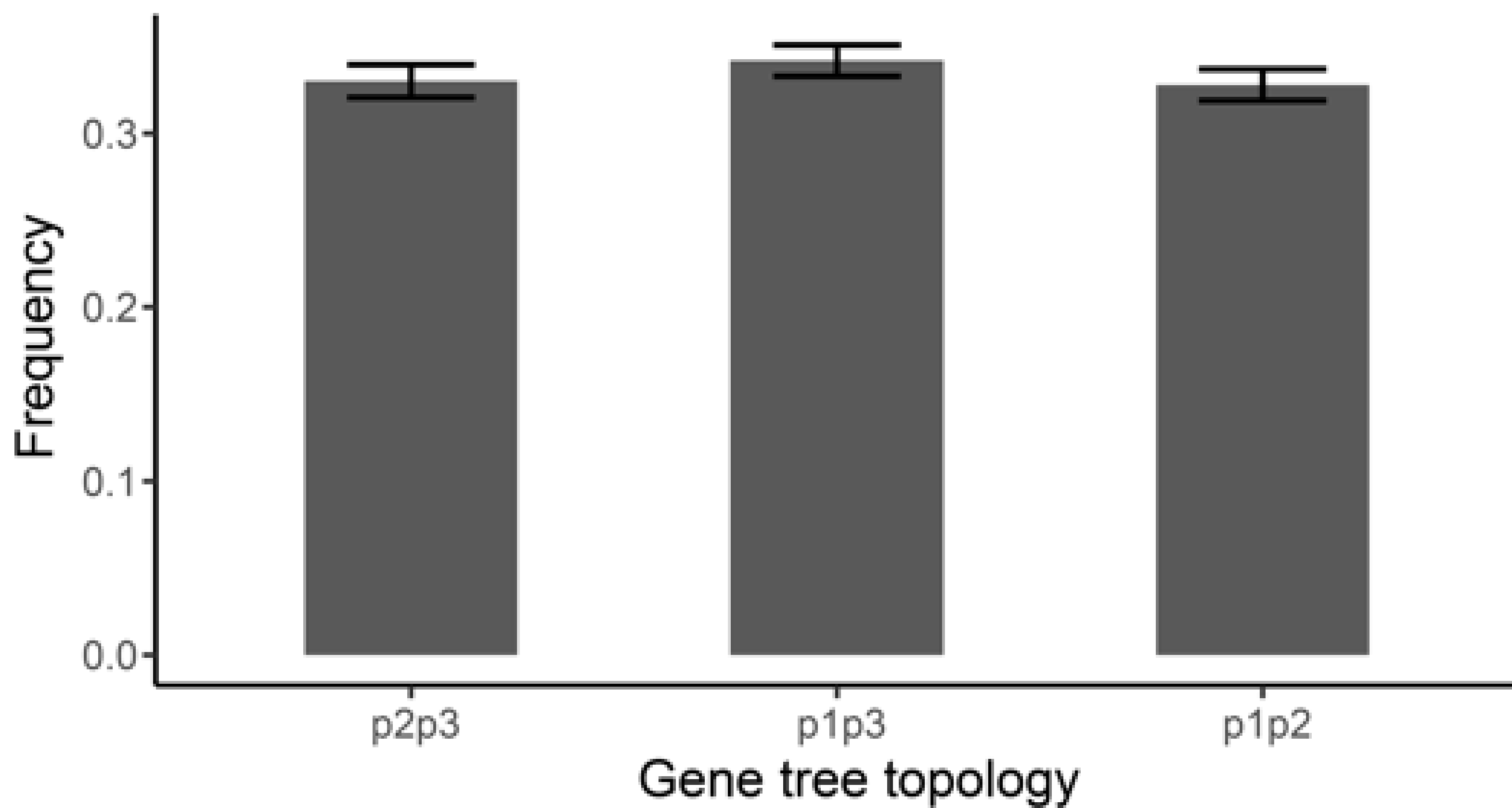

B) Per locus mutation rate =  $3 \times 10^{-5}$

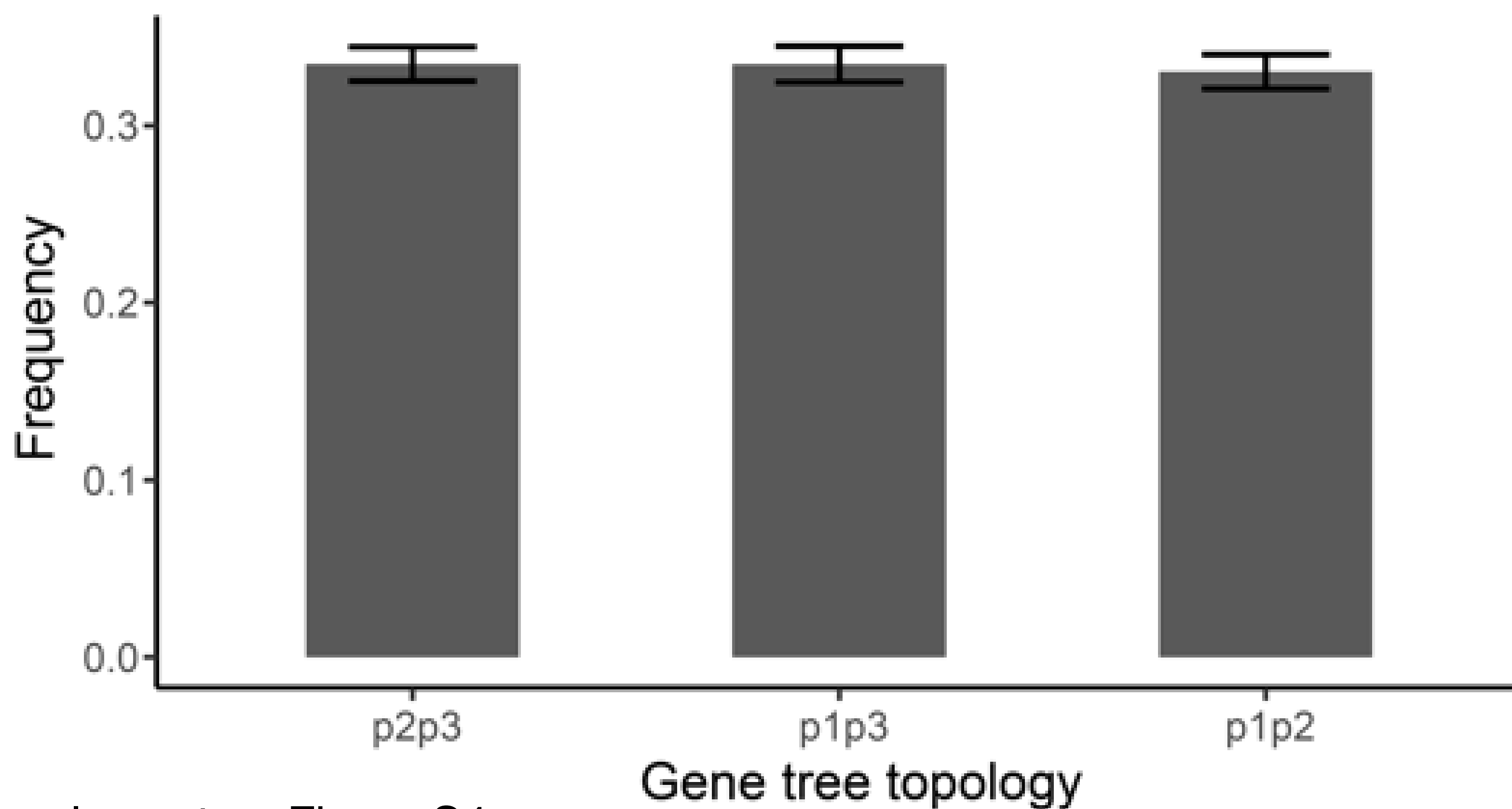

Supplementary Figure S4.

Supplement: S4 Fig — (2020): population size combination “F” with s = −7.5 × 10^−6^ and Δτ = 2,000. Our results show no significant difference in the distribution of gene tree topologies in the presence of negative selection (A). This result holds for simulations in which we increased the per-locus mutation rate by 2 orders of magnitude (B). SLiM3 recipes are available via Data Dryad at https://doi.org/10.5061/dryad.rfj6q577d [22]. Gene tree counts for both simulations, A and B, are available in S1 Data. (PDF) [file pbio.3000954.s004.pdf]

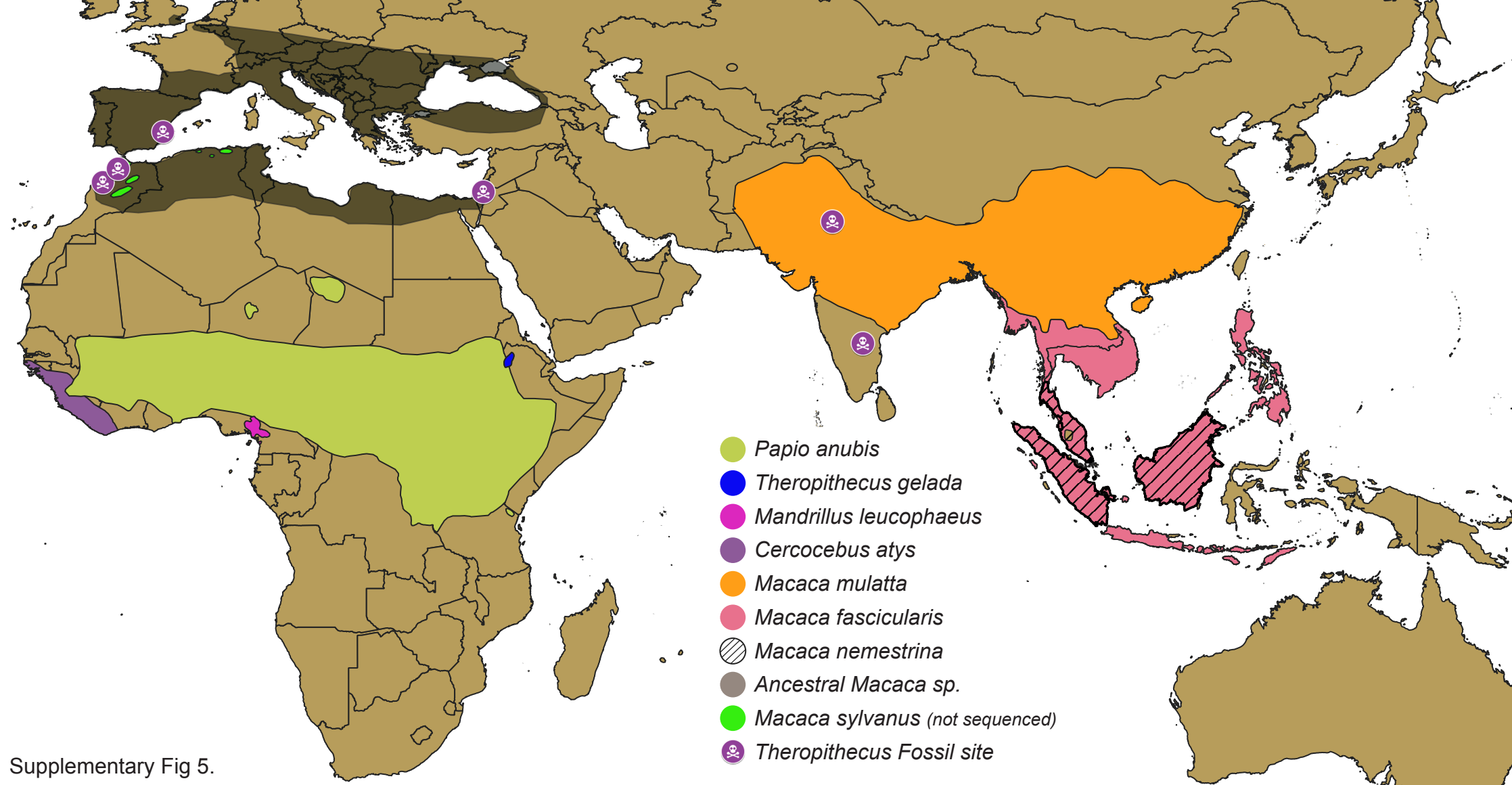

Supplementary Fig 5.

Supplement: S5 Fig — The ancestral Macaca distribution (gray shading) is inferred from Macaca fossil localities in Africa and Europe as reviewed in Roos et al. [106]. The ancestral Macaca distribution likely represents only a fraction of the species range from the late Miocene to the late Pleistocene in Africa and Europe. The contemporary distribution of the African Macaca sylvanus (bright green) is included for reference; the current distribution of Macaca nemestrina is completely contained within that of Macaca fascicularis. Fossil localities for Theropithecus species hypothesized to overlap contemporaneously with various ancestral Macaca are included. Citations for spatial data of extant species: M. nemestrina (Richardson et al., 2008), M. fascicularis (Ong and Richardson, 2008), M. sylvanus (Butynski et al., 2008), Macaca mulatta (Timmins et al., 2008), Theropithecus gelada (Gippoliti et al., 2019), Papio anubis (Kingdon et al., 2008), Cercocebus atys (Oates et al., 2016), and Mandrillus leucophaeus (Oates and Butynski, 2008). Base map was obtained from the public domain map database Natural Earth (http://www.naturalearthdata.com/downloads/). (PDF) [file pbio.3000954.s005.pdf]
