# Supplementary material for: Primate phylogenomics uncovers multiple rapid radiations and ancient interspecific introgression
Source: PLoS Biol. 2020 Dec 3;18(12):e3000954. doi: 10.1371/journal.pbio.3000954 (PMC7738166; doi:10.1371/journal.pbio.3000954)
Supplement: S1 Table — When possible, the most recent version for each genome was used. (DOCX) [file pbio.3000954.s006.docx]

| Species | NCBI Year | Reference | Accession Used this Study |
| --- | --- | --- | --- |
| *Aotus nancymaae* (Ma’s night monkey) | 2015 | [1] | GCF_000952055.2 |
| *Callithrix jacchus* (White-tufted-ear marmoset) | 2010 | [2] | GCF_000004665.1 |
| *Carlito syrichya* (Philippine tarsier) | 2013 | [3] | GCF_000164805.1 |
| *Cebus capucinus imitator* (White-faced sapajou) | 2016 | Dr. Amanda Melin, Washington University St. Louis;  Dr. Shoji Kawamura, University of Tokyo;  Dr. Wesley Warren, McDonnell Genome Institute;  Washington University School of Medicine, Unpublished | GCF_001604975.1 |
| *Cercocebus atys* (Sooty mangabey) | 2015 | [4] | GCF_000955945.1 |
| *Chlorocebus sabaeus* (Green monkey) | 2014 | [5] | GCF_000409795.2 |
| ***Colobus angolensis palliatus* (Black and white colobus)** | **2015** | **This Study** | **GCF_000951035.1** |
| *Gorilla gorilla gorilla* (Western gorilla) | 2006 | [6] | GCF_000151905.2 |
| *Homo sapiens* | 2001 | [7] | GCF_000001405.38 (GRCh38.p12) |
| *Macaca fascicularis* (Crab-eating macaque) | 2011 | [8] | GCF_000364345.1 |
| ***Macaca nemestrina* (Pig-tailed macaque)** | **2015** | **This Study** | **GCF_000956065.1** |
| *Macaca mullata* (Rhesus macaque) | 2006 | [9] | GCF_000772875.2 |
| ***Mandrillus leucophaeus* (Drill)** | **2015** | **This Study** | **GCF_000951045.1** |
| *Microcebus murinus* (Gray mouse lemur) | 2007 | [10] | GCF_000165445.2 |
| *Nomascus leucogenys* (Northern white-cheeked gibbon) | 2010 | [11] | GCF_000146795.2 |
| *Otolemur garnetti* (Small-eared galago) | 2006 | Broad Institute, 2011, Unpublished | GCF_000181295.1 |
| *Pan paniscus* (Pygmy chimpanzee) | 2012 | [12] | GCF_000258655.2 |
| *Pan troglodytes* | 2005 | [13] | GCF_002880755.1 |
| *Papio anubis* (Olive baboon) | 2012 | [14] | GCF_000264685.3 |
| *Piliocolobus tephrosceles* (Ugandan red colobus) | 2017 | University of Oregon, 2017, Unpublished | GCF_002776525.1 |
| *Pongo abelii* (Sumatran orangutan) | 2006 | University of Washington, 2017, Unpublished | GCF_002880775.1 |
| *Propithecus coquereli* (Coquerel’s sifaka) | 2015 | Baylor College of Medicine, 2015, Unpublished | GCF_000956105.1 |
| *Rhinopithecus bieti* (Black snub-nosed monkey) | 2016 | [15] | GCF_001698545.1 |
| *Rhinopithecus roxellana* (Golden snub-nosed monkey) | 2014 | [16] | GCF_000769185.1 |
| *Saimiri boliviensis boliviensis* (Bolivian squirrel monkey) | 2011 | Broad Institute, 2011, Unpublished | GCF_000235385.1 |
| *Theropithecus gelada* (Gelada) | 2018 | University of Washington, 2018, Unpublished | GCF_003255815.1 |
| *Tupaia chinensis* (Chinese tree shrew) | 2013 | [17] | GCF_000334495.1 |
| *Mus musculus* C57BL/6J (House Mouse) | 2002 | [18] | GCF_000001635.26, GRCm38.p4 (Annotation release 106, 2016) |
| *Galeopterus variegatus* (Sunda flying lemur) | 2014 | Washington University, 2014, Unpublished | GCF_000696425.1 |

**S1 Table**. Genomes analyzed in this study with the original NCBI release date, the publication for the reference used, and the accession number for the assembly. When possible the most recent version for each genome was used.

**References**

1. Thomas GWC, Wang RJ, Puri A, Harris RA, Raveendran M, Hughes DST, et al. Reproductive longevity predicts mutation rates in primates. Curr Biol CB. 2018;28: 3193–3197.e5. doi:10.1016/j.cub.2018.08.050

2. Marmoset Genome Sequencing and Analysis Consortium. The common marmoset genome provides insight into primate biology and evolution. Nat Genet. 2014;46: 850–857. doi:10.1038/ng.3042

3. Schmitz J, Noll A, Raabe CA, Churakov G, Voss R, Kiefmann M, et al. Genome sequence of the basal haplorrhine primate *Tarsius syrichta* reveals unusual insertions. Nat Commun. 2016;7: 12997. doi:10.1038/ncomms12997

4. Palesch D, Bosinger SE, Tharp GK, Vanderford TH, Paiardini M, Chahroudi A, et al. Sooty mangabey genome sequence provides insight into AIDS resistance in a natural SIV host. Nature. 2018;553: 77–81. doi:10.1038/nature25140

5. Warren WC, Jasinska AJ, García-Pérez R, Svardal H, Tomlinson C, Rocchi M, et al. The genome of the vervet (*Chlorocebus aethiops sabaeus*). Genome Res. 2015;25: 1921–1933. doi:10.1101/gr.192922.115

6. Scally A, Dutheil JY, Hillier LW, Jordan GE, Goodhead I, Herrero J, et al. Insights into hominid evolution from the gorilla genome sequence. Nature. 2012;483: 169. doi:10.1038/nature10842

7. Church DM, Schneider VA, Graves T, Auger K, Cunningham F, Bouk N, et al. Modernizing reference genome assemblies. PLoS Biol. 2011;9: e1001091. doi:10.1371/journal.pbio.1001091

8. Yan G, Zhang G, Fang X, Zhang Y, Li C, Ling F, et al. Genome sequencing and comparison of two nonhuman primate animal models, the cynomolgus and Chinese rhesus macaques. Nat Biotechnol. 2011;29: 1019–1023. doi:10.1038/nbt.1992

9. Zimin AV, Cornish AS, Maudhoo MD, Gibbs RM, Zhang X, Pandey S, et al. A new rhesus macaque assembly and annotation for next-generation sequencing analyses. Biol Direct. 2014;9: 20. doi:10.1186/1745-6150-9-20

10. Larsen PA, Harris RA, Liu Y, Murali SC, Campbell CR, Brown AD, et al. Hybrid de novo genome assembly and centromere characterization of the gray mouse lemur (*Microcebus murinus*). BMC Biol. 2017;15: 110. doi:10.1186/s12915-017-0439-6

11. Carbone L, Harris RA, Gnerre S, Veeramah KR, Lorente-Galdos B, Huddleston J, et al. Gibbon genome and the fast karyotype evolution of small apes. Nature. 2014;513: 195–201. doi:10.1038/nature13679

12. Prüfer K, Munch K, Hellmann I, Akagi K, Miller JR, Walenz B, et al. The bonobo genome compared with the chimpanzee and human genomes. Nature. 2012;486: 527–531. doi:10.1038/nature11128

13. Chimpanzee Sequencing and Analysis Consortium. Initial sequence of the chimpanzee genome and comparison with the human genome. Nature. 2005;437: 69–87. doi:10.1038/nature04072

14. Rogers J, Raveendran M, Harris RA, Mailund T, Leppälä K, Athanasiadis G, et al. The comparative genomics and complex population history of Papio baboons. Sci Adv. 2019;5: eaau6947. doi:10.1126/sciadv.aau6947

15. Yu L, Wang G-D, Ruan J, Chen Y-B, Yang C-P, Cao X, et al. Genomic analysis of snub-nosed monkeys (*Rhinopithecus*) identifies genes and processes related to high-altitude adaptation. Nat Genet. 2016;48: 947–952. doi:10.1038/ng.3615

16. Zhou X, Wang B, Pan Q, Zhang J, Kumar S, Sun X, et al. Whole-genome sequencing of the snub-nosed monkey provides insights into folivory and evolutionary history. Nat Genet. 2014;46: 1303–1310. doi:10.1038/ng.3137

17. Fan Y, Huang Z-Y, Cao C-C, Chen C-S, Chen Y-X, Fan D-D, et al. Genome of the Chinese tree shrew. Nat Commun. 2013;4: 1426. doi:10.1038/ncomms2416

18. Mouse Genome Sequencing Consortium, Waterston RH, Lindblad-Toh K, Birney E, Rogers J, Abril JF, et al. Initial sequencing and comparative analysis of the mouse genome. Nature. 2002;420: 520–562. doi:10.1038/nature01262
