# Supplementary material for: Primate phylogenomics uncovers multiple rapid radiations and ancient interspecific introgression
Source: PLoS Biol. 2020 Dec 3;18(12):e3000954. doi: 10.1371/journal.pbio.3000954 (PMC7738166; doi:10.1371/journal.pbio.3000954)
Supplement: S4 Table — * denotes species sequenced this study. (DOCX) [file pbio.3000954.s009.docx]

| Species | #Gap/Ambiguity | Percentage |
| --- | --- | --- |
| *Macaca mulatta* | 71,090 | 4.04% |
| *Rhinopithecus roxellana* | 80,328 | 4.56% |
| *Chlorocebus sabaeus* | 88,366 | 5.02% |
| *Macaca nemestrina** | 88,650 | 5.03% |
| *Pongo abelii* | 89,290 | 5.07% |
| *Macaca fascicularis* | 97,942 | 5.56% |
| *Pan troglodytes* | 101,013 | 5.74% |
| *Cebus capucinus imitator* | 101,636 | 5.77% |
| *Cercocebus atys* | 102,521 | 5.82% |
| *Papio anubis* | 110,028 | 6.25% |
| *Aotus nancymaae* | 110,052 | 6.25% |
| *Microcebus murinus* | 114,128 | 6.48% |
| *Piliocolobus tephrosceles* | 115,512 | 6.56% |
| *Rhinopithecus bieti* | 119,118 | 6.76% |
| *Propithecus coquereli* | 120,633 | 6.85% |
| *Theropithecus gelada* | 128,355 | 7.29% |
| *Otolemur garnettii* | 128,999 | 7.33% |
| *Saimiri boliviensis* | 143,099 | 8.13% |
| *Gorilla gorilla* | 143,787 | 8.17% |
| *Callithrix jacchus* | 148,292 | 8.42% |
| *Pan paniscus* | 155,109 | 8.81% |
| *Homo sapiens* | 159,941 | 9.08% |
| *Colobus angolensis palliatus** | 177,747 | 10.09% |
| *Nomascus leucogenys* | 188,893 | 10.73% |
| *Galeopterus variegatus* | 199,749 | 11.34% |
| *Mus musculus* | 201,118 | 11.42% |
| *Tupaia chinensis* | 201,166 | 11.42% |
| *Mandrillus leucophaeus** | 204,583 | 11.62% |
| *Carlito syrichta* | 323,435 | 18.37% |

**S4 Table**. Gaps/Ambiguities by species, and as a percentage of total alignment length.

* denotes species sequenced this study.
