# Supplementary material for: Primate phylogenomics uncovers multiple rapid radiations and ancient interspecific introgression
Source: PLoS Biol. 2020 Dec 3;18(12):e3000954. doi: 10.1371/journal.pbio.3000954 (PMC7738166; doi:10.1371/journal.pbio.3000954)
Supplement: S5 Table — Each dataset was analyzed twice until node age estimates converged (15–25k steps) using a log-normal auto-correlated model [139]. Datasets are available via Data Dryad at https://doi.org/10.5061/dryad.rfj6q577d [22]. (DOCX) [file pbio.3000954.s010.docx]

| Dataset | Length (bp) |
| --- | --- |
| Dataset 1 | 39,675 |
| Dataset 2 | 40,908 |
| Dataset 3 | 41,703 |
| Dataset 4 | 42,137 |
| Dataset 5 | 40,227 |
| Dataset 6 | 35,898 |
| Dataset 7 | 36,750 |
| Dataset 8 | 35,893 |
| Dataset 9 | 37,805 |
| Dataset 10 | 42,748 |

**S5 Table**. Lengths for each 40-locus concatenated alignment used in the molecular dating analyses. Each dataset was analyzed twice until node age estimates converged (15-25k steps) using a log-normal auto-correlated model [1]. Datasets are available via Data Dryad at <https://doi.org/10.5061/dryad.rfj6q577d> [2].

**References**

1. Thorne JL, Kishino H, Painter IS. Estimating the rate of evolution of the rate of molecular evolution. Mol Biol Evol. 1998;15: 1647–1657.

2. Vanderpool D. Data from: Primate phylogenomics uncovers multiple rapid radiations and ancient interspecific introgression. In: Dryad Digital Repository. [Internet]. 2020. Available: https://doi.org/10.5061/dryad.rfj6q577d
