# Supplementary material for: Primate phylogenomics uncovers multiple rapid radiations and ancient interspecific introgression
Source: PLoS Biol. 2020 Dec 3;18(12):e3000954. doi: 10.1371/journal.pbio.3000954 (PMC7738166; doi:10.1371/journal.pbio.3000954)
Supplement: S6 Table — Node numbering corresponds to the numbering in Fig 3. Median underflow/overflow for each calibration was calculated from 20 independent runs performed on 10 datasets (2 runs per dataset). (DOCX) [file pbio.3000954.s011.docx]

| Node (Descendent Lineages) | Minimum Age MYA (citation) | Median Underflow (stdev) | Maximum Age (MYA) | Median Overflow (stdev) |
| --- | --- | --- | --- | --- |
| Node 5 (Simiiformes, Tarsiiformes) | 43 [1–3] | 0 (1.48) | NA | NA |
| Node 6 (Strepsirrhini, Haplorrhini) | 55.8 [4,5] | 0.87% (0.43) | 65.8 [6] | 3.37% (1.36) |
| Node 7 (Dermoptera, Primates) | 61  [6,7] | 5% (3.26) | 165 [6] | 0 (0.29) |
| Node 9 (Lorisiformes, Lemuriformes) | 38 [8] | 0.15% (0.65) | 56 [5,6] | 0.19% (0.39) |
| Node 11 (Catarrhini) | 25 [9] | 2% (1.48) | 34 [6] | 0.34% (2.58) |
| Node 13 (*Pongo, Homo*) | 14 [10] | 0% (0.79) | 34 [6] | 0% |
| Node 15 (*Homo, Pan*) | 5.7 [11] | 0.12% (0.62) | 10 [6] | 3.69% (2.58) |
| Node 22 (*Macaca, Papio*) | 7 [12] | 1.28% (7.16) | NA | NA |

**S6 Table**. Fossil calibrations employed in this study. Node numbering corresponds to the numbering in Figure 3. Median underflow/overflow for each calibration was calculated from 20 independent runs performed on 10 datasets (2 runs per dataset).

**References**

1. Franzen JL, Gingerich PD, Habersetzer J, Hurum JH, von Koenigswald W, Smith BH. Complete primate skeleton from the Middle Eocene of Messel in Germany: morphology and paleobiology. PloS One. 2009;4: e5723. doi:10.1371/journal.pone.0005723

2. Perelman P, Johnson WE, Roos C, Seuánez HN, Horvath JE, Moreira MAM, et al. A molecular phylogeny of living primates. PLoS Genet. 2011;7: e1001342. doi:10.1371/journal.pgen.1001342

3. Poux C, Douzery EJP. Primate phylogeny, evolutionary rate variations, and divergence times: a contribution from the nuclear gene *IRBP.* Am J Phys Anthropol. 2004;124: 1–16. doi:10.1002/ajpa.10322

4. Bloch JI, Silcox MT, Boyer DM, Sargis EJ. New Paleocene skeletons and the relationship of plesiadapiforms to crown-clade primates. Proc Natl Acad Sci. 2007;104: 1159–1164. doi:10.1073/pnas.0610579104

5. Sigé B, Jaeger J-J, Sudre J, Vianey-Liaud M. *Altiatlasius koulchii* n. gen. et sp., primate omomyidé du Paléocène supérieur du Maroc, et les origines des euprimates. Palaeontogr Abt A. 1990; 31–56.

6. Benton MJ, Donoghue PCJ, Asher RJ, Friedman M, Near TJ, Vinther J. Constraints on the timescale of animal evolutionary history. Palaeontol Electron. 2015;18: 1–106. doi:10.26879/424

7. Clemens WA, Wilson GP. Early Torrejonian mammalian local faunas from northeastern Montana, USA. Mus North Ariz Bull. 2009;65: 111–158.

8. Seiffert ER, Simons EL, Attia Y. Fossil evidence for an ancient divergence of lorises and galagos. Nature. 2003;422: 421–424. doi:10.1038/nature01489

9. Stevens NJ, Seiffert ER, O’Connor PM, Roberts EM, Schmitz MD, Krause C, et al. Palaeontological evidence for an Oligocene divergence between Old World monkeys and apes. Nature. 2013;497: 611–614. doi:10.1038/nature12161

10. Raaum RL, Sterner KN, Noviello CM, Stewart C-B, Disotell TR. Catarrhine primate divergence dates estimated from complete mitochondrial genomes: concordance with fossil and nuclear DNA evidence. J Hum Evol. 2005;48: 237–257. doi:10.1016/j.jhevol.2004.11.007

11. Brunet M, Guy F, Pilbeam D, Mackaye HT, Likius A, Ahounta D, et al. A new hominid from the Upper Miocene of Chad, Central Africa. Nature. 2002;418: 145–151. doi:10.1038/nature00879

12. Steiper ME, Young NM, Sukarna TY. Genomic data support the hominoid slowdown and an Early Oligocene estimate for the hominoid–cercopithecoid divergence. Proc Natl Acad Sci. 2004;101: 17021–17026. doi:10.1073/pnas.0407270101
