# Supplementary material for: Primate phylogenomics uncovers multiple rapid radiations and ancient interspecific introgression
Source: PLoS Biol. 2020 Dec 3;18(12):e3000954. doi: 10.1371/journal.pbio.3000954 (PMC7738166; doi:10.1371/journal.pbio.3000954)
Supplement: S7 Table — Node numbers correspond to the numbering in Fig 3. The 95% HPD intervals were calculated by averaging the minimum and maximum of the 95% HPD interval for each dating run. HPD, highest posterior density. (DOCX) [file pbio.3000954.s012.docx]

| Node Number | Mean Node Age (stdev) | Mean 95% Credibility Intervals |
| --- | --- | --- |
| 1 | 16.97 (1.20) | 12.91 - 21.75 |
| 2 | 15.12 (1.17) | 11.25 - 19.59 |
| 3 | 17.21 (1.19) | 13.09 - 22.00 |
| 4 | 36.57 (0.59) | 32.16 - 41.41 |
| 5 | 59.01 (0.66) | 53.77 - 63.38 |
| 6 | 61.68 (0.53) | 56.23 - 65.90 |
| 7 | 66.57 (0.91) | 60.37 - 72.20 |
| 8 | 68.92 (1.69) | 62.10 - 75.75 |
| 9 | 47.74 (0.99) | 40.44 - 54.23 |
| 10 | 31.04 (2.02) | 20.95 - 40.86 |
| 11 | 28.41 (0.92) | 25.06 - 32.84 |
| 12 | 21.38 (0.74) | 17.96 - 25.47 |
| 13 | 19.19 (0.80) | 15.93 - 22.99 |
| 14 | 10.94 (0.73) | 8.48 - 13.15 |
| 15 | 8.54 (0.45) | 6.48 - 10.05 |
| 16 | 3.27 (0.82) | 2.18 - 4.54 |
| 17 | 16.79 (1.30) | 13.45 - 20.98 |
| 18 | 12.24 (1.32) | 9.16 - 16.17 |
| 19 | 10.14 (1.35) | 7.35 - 13.76 |
| 20 | 3.59 (0.95) | 2.22 - 5.77 |
| 21 | 12.83 (1.48) | 9.85 - 16.73 |
| 22 | 9.99 (1.57) | 7.46 - 13.44 |
| 23 | 5.65 (1.33) | 3.85 - 8.28 |
| 24 | 4.01 (1.28) | 2.61 - 6.06 |
| 25 | 8.53 (1.45) | 6.22 - 11.71 |
| 26 | 7.33 (1.49) | 5.21 - 10.22 |
| 27 | 5.85 (1.06) | 4.05 - 8.40 |

**S7 Table**. Mean node age for 20 independent PhyloBayes dating runs. Node numbers correspond to the numbering in Fig 3. The 95% HPD intervals were calculated by averaging the minimum and maximum of the 95% HPD interval for each dating run.
