# Supplementary material for: Primate phylogenomics uncovers multiple rapid radiations and ancient interspecific introgression
Source: PLoS Biol. 2020 Dec 3;18(12):e3000954. doi: 10.1371/journal.pbio.3000954 (PMC7738166; doi:10.1371/journal.pbio.3000954)
Supplement: S8 Table — Branches tested correspond to the labeled branches in Fig 3. After correcting for multiple comparisons (Dunn–Šidák, P = 0.00301), 3 internal branches and 8 quartets were found to have significant Δ values, indicating a likely introgression event. (DOCX) [file pbio.3000954.s013.docx]

| (((*P_1_*,*P_2_*),*P_3_*),*O*) (Branch #, Figure 3) | Gene Trees | DF1/DF2 Counts, *P*-value | Significant at Dunn-Šidák *P* = 0.00301 |
| --- | --- | --- | --- |
| (((*Carlito, M.* *mulatta*), *Otolemur*),*Galeopterus*) (# 5) | 4,908 | 1170/1150, *p* = 0.687 | ⮽ |
| (((*Homo, Pongo*), *Nomascus*), *M.* *mulatta*) (# 13) | 4,326 | 913/823, *p* = 0.030 | ⮽ |
| (((*Homo, Chimp*), *Gorilla*), *Nomascus*) (# 15) | 4,099 | 703/635, *p* = 0.0625 | ⮽ |
| (((*Chlorocebus, Papio*), *Colobus*), *Homo*) (# 51) | 6,358 | 709/646, *p* = 0.0854 | ⮽ |
| (((*M.* *mulatta, M.* *nemestrina*),*Theropithecus*), *Colobus*) (# 23) | 5,109 | 216/194, *p* = 0.2710 | ⮽ |
| (((*M.* *mulatta, M. fascicularis*), *M.* *nemestrina*), *Colobus*) (# 24) | 3,579 | 708/496, *p* = 1.373e -09 | 🗸 |
| (((*Papio, Cercocebus*), *M.* *nemestrina*), *Colobus*) (# 25)  (((*Papio, Cercocebus*), *M.* *mulatta*), *Colobus*)  (((*Papio, Cercocebus*), *M. fascicularis*), *Colobus*)  (((*Papio, Mandrillus*), *M.* *nemestrina*), *Colobus*)  (((*Papio, Mandrillus*), *M.* *mulatta*), *Colobus*)  (((*Papio, Mandrillus*), *M. fascicularis*), *Colobus*)  (((*Theropithecus, Cercocebus*), *M.* *nemestrina*), *Colobus*)  (((*Theropithecus, Cercocebus*), *M.* *mulatta*), *Colobus*)  (((*Theropithecus, Cercocebus*), *M. fascicularis*), *Colobus*)  (((*Theropithecus, Mandrillus*), *M.* *nemestrina*), *Colobus*)  (((*Theropithecus, Mandrillus*), *M.* *mulatta*), *Colobus*)  (((*Theropithecus, Mandrillus*), *M. fascicularis*), *Colobus*) | 4,290  4,278  4,288  4,386  4,360  4,375  4,323  4,303  4,305  4,306  4,277  4,261 | 863/821, *p* = 0.2998  838/809, *p* = 0.4726  867/808, *p* = 0.1428  1119/764, *p* = 2.220e -16  1099/739, *p* = 0.0  1125/735, *p* = 0.0  993/766, *p* = 6.679e -08  975/736, *p* = 4.958e -09  1000/748, *p* = 3.020e -09  888/806, *p* = 0.0440  872/759, *p* = 0.0059  866/771, *p* = 0.01 | ⮽  ⮽  ⮽  🗸  🗸  🗸  🗸  🗸  🗸  ⮽  ⮽  ⮽ |
| (((*Papio, Theropithecus*), *Cercocebus*), *Colobus*) (# 27) | 5,605 | 912/569, *p* = 0.0 | 🗸 |

**S8 Table.** Quartets used to test for significant ∆ values for internal branches of the primate tree. Branches tested correspond to the labeled branches in Figure 3. After correcting for multiple comparisons (Dunn-Šidák, *P* = 0.00301), three internal branches and 8 quartets were found to have significant ∆ values, indicating a likely introgression event.
