# Supplementary material for: Primate phylogenomics uncovers multiple rapid radiations and ancient interspecific introgression
Source: PLoS Biol. 2020 Dec 3;18(12):e3000954. doi: 10.1371/journal.pbio.3000954 (PMC7738166; doi:10.1371/journal.pbio.3000954)
Supplement: Attachment — Submitted filename: Response_to_Reviewers.docx [file pbio.3000954.s015.docx]

We thank the editor and reviewers for their comments. As detailed below, we have clarified all of the issues raised by the reviewers, as well as carrying out many new analyses. Some of the new things in the manuscript include:

- An extensive supplementary dataset.
- Re-estimated divergence times using the gene trees with highest concordance.
- Re-estimated concordance measures using the longest genes.
- Re-estimated concordance measures using an additional outgroup.
- An examination of the relationship between depth in the tree and concordance.
- Extensive simulations to show that selection does not lead to discordance.

Many of these analyses are included in a new section of the manuscript entitled “Sources of Gene Tree Discordance.” We have also carried out analyses of rare genomic changes, finding good evidence for discordance in several contentious nodes. However, as detailed in our responses below, technical limitations with these analyses lead us to not include them in the manuscript.

While these new analyses have led us to include several important caveats to our study, all of the major results and conclusions remain. We hope that the new manuscript meets the high standards of your journal.

*****************************************************
REVIEWERS' COMMENTS:


*Reviewer #1:
[identifies himself as Matt Pennell]

This is a really exceptional contribution to phylogenetics. The analyses were well-thought and thoroughly done -- I feel very confident in your conclusions. And the writing is clear throughout; I appreciated the mix of methodological details with natural history/biological context. I anticipate that I will add this to the reading list for my phylogenetics class next year as a superb example of the power of modern data and approaches to phylogenomics.* 

We thank the reviewer for this very positive response to our study.

*I have only a few minor points that I think might be worth addressing before publication.* 

*Line 150: I think this is slightly confusing since the underlying gene trees used in the ASTRAL analysis were also estimated with IQTREE2. The way it is written it makes it seem that IQTREE2 was used only for the concatenated analysis.*

We agree that this was confusing, and have removed this sentence. We have instead expanded this to two separate sentences for clarity (Line 183):

“We inferred 1,730 individual gene trees from nucleotide alignments using maximum likelihood in IQ-TREE 2 [38], and then inferred a species tree using these gene tree topologies as input to ASTRAL III ([39]; Materials and Methods)”

and (Line 188):

“We also used IQ-TREE to carry out a maximum likelihood analysis of the concatenated nucleotide alignment (a topology we refer to as “ML-CONCAT”).”

*Line 163: I will admit my ignorance and say that I didn't know what the phrase "maximal local posteriors" meant and had to look it up. Given that this is a relatively new/uncommon term, I think it might be worth devoting an extra clause or sentence to explaining.*

We have updated the sentence to explain this term (Line 194):

“All branches of the ML-ASTRAL species tree are supported by maximum local posteriors, the default support values provided by ASTRAL III [40], except for the branch that defines *Aotus* as sister to the *Saimiri*+*Cebus* clade (0.46 local posterior probability).”

*Line 167-168: I would really appreciate a few additional sentences giving an outline of this controversy. As it stands now, it is really hard to know what all the fuss is about. In a later section, you do a really great job discussing the controversies over the placement of tarsiers and I think something similar would be very useful here.* 

We have expanded this section to say (Line 200):

“There has been some contention as to the placement of the mammalian orders Scandentia (treeshrews) and Dermoptera (colugos) [41–50]. The controversy concerns whether Dermoptera is sister to Primates, Scandentia is sister to Primates, or Dermoptera and Scandentia are sister groups.”

*Line 237-238: I realize that this is a throwaway line/citation but I think the evidence for the association between primate diversification and global temperature is pretty weak in my view. I guess it is fine to speculate but this is presented as a clear fact. Given how precise the rest of the paper is, this statement is uncharacteristically flippant.*

We have removed the statement about the impact of climate on this radiation.

*Line 264: I understand what you are getting at here but I think it is worth spelling out the argument for using parsimony in this case; it is not really clear from the text alone why you think parsimony is going to be a more consistent estimator than ML here.*

We now try to be explicit about why we need to use parsimony in such cases (Line 306):

“The trees produced from concatenated alignments can be biased in situations with high ILS when maximum likelihood is used for inference, but this bias does not affect parsimony methods [21,72].”

*Line 294: Do you have any (more direct) evidence that intralocus recombination is an issue here? I think it may well be true but I think this is a stretch to infer from the increased concordance for the MP trees.*

Although we don’t have evidence of recombination from this particular dataset, we do have evidence for an effect of intralocus recombination from a subset of the taxa sampled here. We previously showed (Mendes *et al.* 2019) that for the ((Human,Chimp)Gorilla) tree there is much more consistency in inferred phylogenetic relationships within exons than between exons (a similar pattern was also found by Scornavacca and Galtier 2017). Such an effect should only be due to recombination within genes.

We have changed the sentence slightly to reference this previous analysis (Line 336):

“The increased number of concordant gene trees using parsimony suggests that the gene trees inferred using ML may well have been suffering from the biases of concatenation when multiple trees are brought together (as observed in the Great Apes, [74]), reducing the observed levels of concordance.” *Line 310: I understand why you had to choose small subsets of the data for the dating analyses but I am wondering what the justification for selecting genes at *random*. Given all the excellent work you've done to determine the branching pattern, I am thinking that it might be worth leveraging this information to pick genes for the dating analyses to reduce the topological noise. Maybe this is a bad idea (and please tell me if it is) but if you wanted to estimate divergence times, why not select randomly from genes that matched the species topology??*

This is a good point. While none of the gene trees in our dataset match the species tree perfectly, we calculated Robinson-Foulds distances for each gene tree to find the top 20 loci with the smallest RF distances to the species tree. These loci were then concatenated and used for a new dating analysis. Possibly as expected (due to the small amount of bias introduced by discordant trees), these new results do not lead to dates that differ significantly from those obtained by averaging over randomly chosen genes. We have added these results to Table 2, and have added new text explaining them (Line 474):

“However, it may be that using genes that have gene trees most similar to the topology being dated will reduce bias caused by concatenation [74]. To determine whether using concordant loci has an impact on the estimated dates, we constructed an eleventh dataset consisting of ~43 kb from the 20 loci most similar to the species tree in Figure 1 (as determined by Robinson-Foulds distances). There was no consistent difference in the dates estimated with this dataset (“Concord” entries in Table 2).

*Line 333: Could you please note in the MS why you didn't include most of the fossils used by Herrera et al. Were most of the ones you didn't use associated with tips rather than nodes.*

The vast majority of the fossils used in Herrera *et al.* are Strepsirrhine fossils. We only had three Strepsirrhines in our study, with only one Strepsirrhine fossil from their study applicable to the taxa sampled here. We used this fossil in our study, as well as the remaining Anthropoid fossils used in Herrera *et. al.* when possible.

We now note this in the manuscript, starting Line 462:

“However, our estimate is in good agreement with Herrera *et al.* [32], who used 34 fossils representing extinct and extant lineages (primarily Strepsirrhines) to infer divergence times among primates, concluding that the split occurred approximately 64 mya. Despite limited overlap in taxon sampling, one similarity between our study and that of Herrera *et al.* is that we have both used the maximum constraint of 65.8 my on the ancestral primate node suggested by Benton *et al.* [90], which likely contributes to the more recent divergence.” *Line 418: I do not think that the line about PhyloNet adds anything to the paper. Who knows what to make of the failure to converge?*

While we agree with your assessment that this particular result does not add much, it does demonstrate the difficulty in disentangling the patterns of introgression in this clade. As we received a request from another reviewer to add analyses to the PhyloNet one, we have left this statement in the manuscript, but now with further context.

*Again, congratulations on an outstanding achievement. 


Reviewer #2:

Vanderpool et al. analyze primate genomes (including three new ones) to assess phylogenetic relationships, incomplete lineage sorting (ILS), and introgression among divergent lineages.  The paper is generally well done and well written, and the conclusions generally follow from the analyses done by the authors.  My primary requirement for revision is to simply post all relevant tree and data files and not just some of these, an essential revision.  The impact of the work will be high I think, and otherwise, the statistical analyses are high quality, and the supplementary information and figures are fine.* 

Thank you for your kind comments. The point regarding the posting of additional supplementary material is well taken. We address this fully in point #21 below.

*The authors also might consider the following in revising their manuscript:

1) line 85.  The authors note that, " Compared to recent hybridization, introgression that occurred between two or more ancestral lineages (represented by internal branches on a phylogeny) is difficult to detect." Introgression involving completely extinct side branches in these trees might also be a problem, as is hybridization with extant lineages for which genomes have not been published.  Is there any way to account for this type of introgression as well?  Authors could comment on this point either way, as I fear that sometimes these types of hybridization events can get lost in the sauce of comparative genomic studies.*

This is a good point. Most methods for detecting introgression cannot determine whether the donor lineage is extant or extinct, and both cases can be detected. We now address this issue (Line 619):

“More insidiously, especially for events further back in time, extinction of the descendants of hybridizing lineages will make it harder to detect introgression (though extinction of donor lineages is less of a problem than extinction of lineages receiving migrants).”

*2) Table 1.  What are the contig N50s for each assembly?  The genome sequencing and assembly methods in the methods section could be fleshed out a bit more than the current terse text.  It is not my specialty, but the description here seems a bit minimal, and it might be difficult for the reader to understand exactly how the genomes were assembled with long reads or without in one case, if I am reading things correctly?*

We have now included the contig N50s in Table 1. The complete information on all 29 assemblies is available in Table S2, including hyperlinks to the NCBI webpages. Information regarding the assembly software and methods are also included on the NCBI pages. *Macaca nemestrina* was the only genome assembled using long-read data. As no special strategy differing from other published genome assemblies was performed, extraneous details were omitted.

*3) line 188.  The authors note regarding conflicts among gene trees at a node that, "...the concordance factors also indicate that a majority of individual topologies have histories that differ from the estimated species tree."  But, most of this conflict, I would bet, is not due to different gene 'histories', and much of this conflict is surely due simply to gene tree reconstruction error.  For example, at the node that groups the tarsier (Carlito) with Simiiformes, the authors report 60% of genes conflicting, but in analyses of retroposon insertions more than a 100 such rare genomic events support this node with absolutely zero conflict. Furthermore, simulations have shown for similar situations that there is extensive gene tree reconstruction error for fairly short internodes that are more than 50 million years old.  So, I do not agree with the authors' interpretation here.  The authors acknowledge this later on line 198, but I do not see any necessity in saying one thing and then ten lines later reversing course.  Same for homoplasious sites versus sites that are incongruent due to ILS or introgression; it is a challenge to distinguish these from each other after a certain level of divergence or maybe even for fairly recent divergences such as chimp, human, gorilla.  A recent study based on large insertions shows that such rare genomic changes completely uniformly favor the chimp+human grouping with absolutely zero conflict.*

Multiple important points are made here, some of which we address further below.

First, so as to be more circumspect about the causes of discordance, we changed the sentence highlighted here to (Line 223):

“…the concordance factors also indicate that a majority of gene tree topologies differ from the estimated species tree.”

Second, we have included a new section in the manuscript: “Sources of Gene Tree Discordance.” In this section we now carry out multiple novel analyses, attempting to distinguish between biological and technical causes of discordance.

One analysis (Line 389) examines the relationship between measures of concordance and depth in the tree. As deeper nodes are expected to be more strongly affected by error, we look for a correlation between concordance factors and the estimated age of each bifurcation in the tree. We found no significant correlation of gCFs with node age (r^2^=0.009), but did find a negative correlation between sCFs and node age (r^2^=0.2998). We therefore conclude (Line 395):

“The negative correlation found between sCFs and node age is consistent with the expectation that substitutions occurring on deeper branches of the tree are more likely to suffer from the effects of multiple substitutions (homoplasy). While there may still be technical factors affecting gCFs, true discordance throughout the tree is high enough to mask any such effect.”

Another of the new analyses re-calculates concordance factors using gene trees and site patterns from the 200 longest alignments in our data set (Line 361), while a third adds an additional outgroup (pika) to a subset of loci to determine whether this has an effect on levels of concordance (Line 372). The results are described in detail in response to reviewer #3 (who suggested these analyses) and also in the new section of the manuscript. In summary, discordance values remain high, though we find that there are some steps we can take to reduce discordance.

Please see below for further analyses addressing the issue of rare genomic events (point #5).

*4) line 204.  As noted just above, for placement of tarsier at least, the authors' argument here on biological causes of gene tree incongruence versus gene tree reconstruction error that causes conflict do not hold up from my perspective.  So, I think a more cautious interpretation is warranted given the uncertainty.*

We address this point fully in our response to points #5 and #12 below. Briefly, after extensive analysis of perfect transposon insertions/deletions using the software GPAC, we find many examples of insertions supporting discordant topologies for the human/chimp/gorilla split in our analysis of both LINEs and Alus. We find a similar pattern when analyzing the human/tarsier/bushbaby split as well (see below).

*5) line 216.  I see here that the authors mention the position of tarsier and note that there is extensive conflict among characters and among gene trees at this node, but this is completely contrary to a published analyses of transposon insertions where more than 100 transposons uniquely support this clade with absolutely no conflicts.  So, if think that the gene tree and character conflicts are actually real, this needs to be reconciled with the published transposon insertion data.  I think it would be a challenge to conclude other than that: 1) the transposon data were made up and are not real (i.e., these other authors cheated), 2) the sequence data analyzed in the current manuscript are homoplasious and gene trees are inaccurately reconstructed, or 3) some sort of weird natural selection drove retroposon insertions to be fixed very rapidly relative to nucleotide substitutions at both silent third codons and selected first and second sites in protein coding genes.  I think the third interpretation would be a challenge to argue for, and the first explanation implies that someone else cheated.*

This is a good point, and indeed the scoring of “rare genomic changes” as characters is becoming increasingly plausible for some datasets. At your suggestion, we looked into this for our dataset but quickly discovered that--despite the continued publication of draft genomes--whole genome alignments with the species of interest still limit this type of analysis. This limit occurs because, as outlined in Kuritzin et al. (2016), a full analysis requires multiple whole-genome comparisons, i.e. full genome alignments to multiple species of interest. Unfortunately, these are not simple to generate, though this point is often not stressed in papers promulgating such approaches.

While all of this means that we cannot carry out a full analysis that would correctly disentangle ILS and introgression, we can do an analysis that attempts to quantify discordance for one of the minor topologies. We used the Genome Presence/Absence Compiler (GPAC) tool (Noll et al., 2015) to find all Alu insertions greater than 50 bp in length across human chromosome 1 for human, tarsier, and bushbaby. This analysis takes advantage of the whole-genome alignments already calculated against the human reference (via the UCSC genome browser). Because genome alignments with tarsier are not available, our analysis cannot detect Alus shared between tarsier and bushbaby. Nevertheless, counting only perfect insertions of Alus in this trio, the results are:

| Number of shared, perfect Alu insertions Human Chr1 | Count |
| --- | --- |
| All Three Taxa (Human, Tarsier, Bushbaby) | 283 |
| Human+Tarsier | 435 |
| Human+Bushbaby | 341 |
| Human Only | 3225 |

Using the KKSC test statistic (Kuritzin et al., 2016) in the one-direction framework to determine the species tree, we see that the tree grouping human+tarsier is strongly favored over the tree uniting human+bushbaby (p=4e-04).

While this taxonomic relationship is what we expect, it is clear that the number of shared insertions between human+bushbaby to the exclusion of tarsier is too high to be explained by homoplasy alone (given the rates of precise parallel insertions (0.01%) and deletions (0.001%) estimated for other primates; Doronina et al., 2019). Although we did not undertake the laborious task of verifying each alignment for all 341 human+bushbaby insertions, a large fraction of these are almost certainly due to ILS.

As to the reason this conflict was not observed by Hartig et al. 2013, it is likely that the stringency of their filtering excluded much of the discordance observed here (as pointed out by the same authors in Kuritzin et al., 2016). For instance, they only identify 19 Alu elements shared by tarsier and human to the exclusion of the Strepsirrhines, while we observe 435 perfect Alus shared between human and tarsiers on human Chr1 alone.

There is also the related issue of a recent paper that did not find any large insertions shared between human and gorilla. To examine the ((Human,Chimp)Gorilla) trio, we carried out a separate analysis of shared Alu and LINE elements on human chromosome 1. Note that this requires a separate whole-genome alignment, as every new trio examined for rare genomic changes requires new alignments. Our results are as follows:

As with the analysis above, because we did not have separate whole-genome alignments to chimpanzee, we cannot assess shared Chimp+Gorilla insertions. However, we hope it is clear that there are quite a number of elements shared discordantly between Human+Gorilla.

Finally, we note that we have previously calculated exact expectations for the number of nucleotide sites discordant due to homoplasy between human and gorilla (Guerrero and Hahn 2018), a calculation that is not dependent at all on inferring discordance from sequence data. We estimate that less than 10% of discordance between these very closely related species is due to homoplasy.

*6) line 222.  The studies that cite conflicting previous placements of the tarsier are ones that analyzed little data.  There are many previous analyses that have strongly supported tarsier plus Simiiformes based on extensive data, and I think none based on lots of data that have supported alternatives, except for analyses where researchers have botched their analyses (e.g., Song et al., 2012).*

The point about extensive data is correct and we now acknowledge this (Line 246):

“Again, our inference of species relationships is consistent with previous genomic analyses [61,62], but also highlights the high degree of discordance in this part of the tree.”

And again (Line 251):

“The complexity of this radiation is likely the reason for low gCF and sCFs (39.5% and 36%, respectively) for the branch leading to Haplorrhini, and perhaps explains why previous studies recovered conflicting resolutions for the placement of tarsiers [31,63,64].”

*7) line 233.  Change "Concatenation Affects Resolution of the New World Monkey Radiation" to "ML Concatenation Affects Resolution of the New World Monkey Radiation"?  The parsimony concatenation seems to agree with the ASTRAL tree here, and only the ML concatenation tree conflicts?  Also, the large ML concatenation tree of Springer et al. (2012) conflicts with the ASTRAL tree from the current study but agrees with ML concatenation analysis of the current study.  Perhaps this is a bias due to ML concatenation but not concatenation generally; what does Perleman et al. ML concatenation support?  If this one also supports the authors' interpretation, this further supports the discussion here.  An interesting empirical result as I have seen few conflicts between ML concatenation and ASTRAL in previous published work.*

As suggested, we have changed this section title to:

"ML Concatenation Affects Resolution of the New World Monkey Radiation"

*8) line 258.  Change "relatively low posterior support" to "very low posterior support".  The PP score is really bordering on minimal?*

We have made this change (Line 289).

*9) line 290.  The inference using parsimony gene trees and ASTRAL is novel and quite interesting.  There has been an affliction in the systematics community in that workers are obsessed with ML methods due to statistical consistency, but of course, this only applies when there is no ILS and there is a single 'gene tree' for all of the data.  Given that there is not unlimited data of this type in any empirical case study, it could be argued that the early simulations that showed parsimony to be lacking/inconsistent are, in the end, bogus and irrelevant.  It is not the first time that arrogance of evolutionary modelers has led to mass confusion in the field.  I fear that the same thing is currently happening on a much grander scale in ecological modeling of COVID death count predictions, but in this latter example, we are dealing with actual life and death. Sad...  But, it is nice that the authors here have actually used a simulation result from the Hahn group and taken the time to run the parsimony trees to reveal this interesting ASTRAL pattern.*

Thank you, we found this pattern interesting as well.

*10) line 294.  Change " biases of concatenation" to " biases of ML concatenation for gene sub-segments with conflicting histories".*

We have now changed the sentence in a slightly different way, but one that we hope captures the idea the reviewer is suggesting (Line 336):

“The increased number of concordant gene trees using parsimony suggests that the gene trees inferred using ML may well have been suffering from the biases of concatenation when multiple trees are brought together (as observed in the Great Apes, [74]), reducing the observed levels of concordance.”

*11) line 358 and above paragraphs.  I found the discussion of divergence times to be reasonable, even-handed, and well stated.  The authors discuss most of the critical issues that impact estimation of dates such as these and explain possible reasons for discrepancies with previous work.*

Thank you for the comments. We have added additional analyses of the most concordant genes to satisfy a comment made by reviewer #1, and were glad to see that this did not significantly change the estimates.

*12) line 370.  The authors note that it is a challenge to infer introgression at deep nodes due to multiple hits.  This is true, but recently Springer et al. have argued that this is a rationale for instead using transposon insertion characters for this purpose, given that these characters are generally thought to be lower-homoplay characters based on their mode of mutation.  Have the authors attempted to use these characters for inferring gene flow among divergent lineages?  If there are enough such characters, I would expect these data to be much more effective than the strategy taken by the authors who used gene trees, as opposed to individual SNPs.  For using gene trees, the challenge is always to determine recombination break points accurately and to deal with different expected average sizes of coalescence-genes for gene trees that support the species tree vs. gene trees that conflict with the species tree (my understanding is that the latter gene trees are generally
expected to be shorter).  It perhaps goes beyond the scope of the current paper to score transposon insertions, but since genomes are assembled, this would not be too much work to do?  If not, it might be worthwhile to at least suggest this strategy for future work in their discussion of this issue, as I think this is the most productive way forward, especially for divergences that are even older, where even more multiple hits are expected and gene tree reconstruction becomes even more challenging.*

We agree with the reviewer that low-homoplasy characters would be especially helpful for testing for introgression. However, as mentioned in response to point #5 above, without generating multi-directional, whole-genome alignments that include representative taxa for the branch of interest, it is not possible to determine whether an asymmetry in the alternative branching pattern at each node exists (cf. Kuritzin et al., 2016). To test for hybridization at each node in the larger tree, these alignments would have to be re-calculated for every internal branch in the tree (i.e. with representatives from each split). In addition, given the boom-bust patterns of activity of transposable elements, appropriate TEs for each split would also have to be independently determined. Unfortunately, all of this could not be done in a timely manner for the species used here.

However, we now do mention this possibility in the text (Line 611):

“One alternative approach to avoid biases in reconstruction could be the use of transposon insertions or other rare genomic changes (cf. [115,116]). Future analyses that compare these different approaches to detecting introgression would be especially useful.”

*13) line 376.  I am guessing that lack of usage of this test is in large part due to the fact that 'genes' like the ones used in this study often span multiple recombination units, so the protein-coding exons strung together here do not likely represent single gene tree histories, but instead many.  The authors acknowledge this somewhat, but I think it is a bigger problem than they maybe let on here?*

It seems as though concatenation of multiple underlying gene trees would make tests of asymmetry using such data conservative, but we admit that we know of no specific results on this point.

*14) line 418.  In addition to PhyloNet, Cecile Ane's group (and others) have developed methods in which introgression pathways are allowed in an ASTRAL-like quartet approach to the ILS+gene flow problem. Might the authors attempt an analysis such as this that accounts for both ILS and gene flow to infer a network using an actual optimality approach (as opposed to PhyloNet which remains a bit mysterious to me in terms of how and why and what it spits out at the end of the process)?*

At the reviewer’s suggestion we used SNaQ in an effort to elucidate the introgression events among the Papionini. Unfortunately, the results were no more informative than those obtained by PhyloNet, with eight equally probable species networks containing two hybrid edges each. Adding more edges simply produced more equi-probable networks.

We have now added the following text mentioning this, just after the results about PhyloNet (Line 567):

“An attempt to infer the network with SNaQ [104] gave similarly ambiguous results.”

*15) line 450.  In terms of caveats, I see that the authors stress more here the issue of recombination, population subdivision, etc., but there is also the problematic aspects of negative selection, which impacts neutral MSC assumptions, in particular because different protein-coding loci are likely under a variety of selective constraints, as well as selective sweeps and diversifying selection.  Proponents of the coalescence approach have tried to avoid these hard truths, but we are starting to see some movement on this front after a recent (and ongoing) exchange in the literature.  For example, He et al. (2019: MBE; "Asymmetric Distribution of Gene Trees Can Arise under Purifying Selection If Differences in Population Size Exist"), but I think the situation is even more dire than this publication lets on...  I predict that it will become very clear that negative selection, of various degrees, will be very problematic for MSC inference and interpretation as time moves on (as argued in Springer and Gatesy, 2016, but denied by "leaders in our field" in Edwards et al., 2016).  Sometimes sets of leaders are really just followers, perhaps?*

The reviewer raises an important point, for multiple reasons. First, to be clear, strong negative selection does not violate the assumption of neutrality. Negative selection is perfectly consistent with neutral evolution of observed variants.

Second, it is hypothetically possible that weak negative selection could bias the distribution of genealogies, and the recent paper by He et al. (2019) does report such an effect. Importantly, if true, these results would have dire consequences for *all* phylogenetic analyses, whether concatenation- or MSC-based. If selection is changing the true underlying distribution of gene trees as much as reported in He et al. (2019), then all phylogenetic methods would infer the wrong species tree.

However, despite the simulation results reported in that paper, there are many years of theoretical results suggesting that weak negative selection does not shift the distribution of gene trees (e.g. Golding 1997; Przeworski et al. 1999; Slade 2000; Williamson and Orive 2002). As He et al. (2019) wrote their own simulation program, with what turns out to be a very non-standard ascertainment scheme for the data analyzed (they threw out 94% of the simulated trees), we decided to run exactly the same simulations using the open-source software, SLiM (all SLiM recipes are also included as a supplemental file). These new simulations required quite a lot of work, and we have added two co-authors who had the necessary expertise to carry out the work accurately.

In summary, we did not find the bias reported by He et al. (2019). Here is the text now reported in the new section of the manuscript (Line 402):

“A recent simulation study [77] reported that negative selection, in combination with large differences in effective population size, can generate strong enough asymmetries in gene tree topologies that the most common topology does not match the species tree. Such an effect, if real, would mislead both gene-tree-based and concatenation-based approaches to species tree inference. However, previous theoretical results predict that there should be no effect of negative selection on the distribution of tree topologies [78–81] and the new results were obtained using custom simulation software. To clarify this issue, we used the open-source simulator SLiM [82] to study non-recombining loci under the most extreme parameters used by He et al. [77] (see Methods). We found no evidence for the bias in gene tree frequencies recently reported (Supplementary Figure S4A). However, we observed fewer than one mutation per locus at the end of our simulations under the parameters exactly replicating He et al. [77], suggesting we may not have generated sufficient deleterious variation to observe the effect. To address this, we simulated the same conditions but with the deleterious mutation rate increased two orders of magnitude, and still did not observe a bias in topology frequencies (Supplementary Figure S4B). Our results therefore indicate that weak negative selection does not generate gene tree discordance, consistent with population genetic theory [78–81].”

We hope that these results help to clarify a possible mistake in the literature.

*16) line 476.  Again here, as long as there are enough active retroelements, I think using these to infer gene flow of ancient lineages solves this problem?*

Again, on this point we agree: retroelement insertions are a great phylogenetic character. However, the time required to perform and curate separate whole-genome alignments across all interior nodes of the tree for multi-directional comparisons is prohibitive at the moment. *17) line 488.  The authors conclude that, "Our analyses reveal how concatenation of genes or even of exons can mislead maximum likelihood phylogenetic inference in the presence of discordance, but also how to overcome the biases introduced by concatenation in some cases."  I am not necessarily convinced that concatenation has failed in any way in this study relative to coalescence methods such as ASTRAL.  A particular brand of this format, ML concatenation, might have failed?*

We have now re-written this sentence to make it clearer that it is solely concatenated ML that is the problem (Line 640):

“Our analyses reveal how concatenation of genes—or even of exons—can mislead maximum likelihood phylogenetic inference in the presence of discordance, but also how to overcome these biases.”  *18) line 545.  The authors note that for concatenation, they did the following: "Q-TREE v2-rc1 was used with all 1,730 aligned loci to estimate a maximum likelihood concatenated (ML-CONCAT) tree with an edge-linked, proportional-partition model".  It might be best for those who have not used IQ-TREE to describe exactly what is entailed in such an analysis.  Was a single substitution model used for the entire concatenation, or were separate substitution models used for each gene in the overall alignment as in the coalescence analyses?  Was just one set of branch lengths used, or were different genes allowed to have different branch lengths?  Were different codon positions given different branch lengths and/or substitution models?*

The following text was added so that the two sentences now read (Line 700):

“IQ-TREE v2-rc1 was used with all 1,730 aligned loci to estimate a maximum likelihood concatenated (ML-CONCAT) tree with an edge-linked, proportional-partition model and 1,000 ultrafast bootstrap replicates [38,131,132]. This strategy uses ModelFinder [133] to automatically find the best-fit model for each ortholog alignment (partition). Branch lengths are shared between partitions, with each partition having its own rate that rescales branch lengths, accommodating different evolutionary rates between partitions.” *19) line 575.  Why just use four taxa here.  This would seem to increase the probability of long branch artifacts as opposed to more complete sampling of taxa that will subdivide long branches?  This type of sampling might in fact lead to asymmetry in results, rather than provide information on that asymmetry?  How do results, for asymmetry, differ when including all taxa (as in the initial estimates that focused on seven nodes) versus the redos with just four taxa that the authors seem to prefer?  Using four taxa automatically gets rid of conflict by removing lineages perhaps and increases the number of relevant loci, but perhaps this is sort of sweeping real issues under the rug?*

These are all very recent divergences, with essentially no chance for artifacts due to long-branch attraction.

*20) line 584.  Given that the authors note much evidence for introgression and also argue that concatenation can be misleading, it is potentially problematic that the clock analyses are all based on concatenation, which would seem to offer distortions.  The clock methods used do not take extensive introgression or ILS into account.  Perhaps the authors should qualify that ILS and introgression could cause distortions in their concatenated clock analyses (perhaps too old dates due to deep coalescence or perhaps too shallow dates due to introgression, etc.).  The authors could run *BEAST which takes ILS into account to get divergence times, as the authors think this is a concern, but note that this coalescence program, like all coalescence methods, are also deeply impacted by introgression (just like concatenation).  The authors naively seem to think that introgression does not impact standard coalescence methods, but this is certainly not the case, in particular for what are
considered by some the best methods (i.e., *BEAST, which has a least common denominator problem with outlier genes as noted in the original description of the method).  The authors should also note how genes were modeled in the concatenated IQ-TREE analyses relative to the concatenated clock analyses.  Again, was each gene given a different model or one model for all genes?*

The reviewer’s concerns regarding variation among loci resulting in different divergence times is valid. This is one reason why we analyzed 10 independent datasets consisting of ~40,000 bp each, to demonstrate that sampling enough loci (~40/dataset; 400 total) results in very similar outcomes for the estimated divergence times. Using *BEAST to estimate divergence times based on these 10 datasets of ~40 kb each (and running each analysis twice to ensure convergence) would require a prohibitively long time.

Another way to address this issue, at least in part, is to use genes that have gene trees concordant with the species tree being dated. In response to a similar question by reviewer #1 we performed an analysis using genes with gene trees that most closely matched the species tree (as determined by using Robinson-Foulds differences). The results of this analysis can be viewed in the response to Reveiwer #1 above, but the estimated dates from the concordant-gene analysis are very similar to those obtained in the analysis of the 10 other analyses.

*21) line 608.  The authors note that, " Single-copy ortholog alignments of CDS sequences produced using Guidance2 and the Nexus formatted dated species tree phylogeny are available through DataDryad (*[*https://doi.org/10.5061/dryad.rfj6q577d*](https://doi.org/10.5061/dryad.rfj6q577d)*)."  The authors should also provide, optimal gene trees, bootstrap consensus gene trees, and concatenated matrixes (with gene partitions) used in their IQ-TREE and clock analyses so that their analyses can be replicated by others if need be.  This is perhaps my strongest comment on revision.  Hard to tell what was done unless all of this material is posted for other scientists to see. So, this revision is essential from my perspective.*

The point regarding the posting of additional supplementary material is a fair one. We included the alignments and species tree file in the original submission as well as the exact commands used to generate the gene trees and species trees from these. We anticipated that interested parties could reproduce the gene trees and ASTRAL trees very quickly using these alignments and commands, obviating the need to post additional files.

However, the reviewer is correct that posting these data directly removes a substantial burden on those wishing to reproduce our results. We have therefore provided these data in the revised submission.

The following files are now available in the Dryad respository:

- 1730_ALIGNMENT_CONCAT.paup.nex – Concatenated alignment with PAUP	block of commands used to generate the parsimony concatenated tree.
- 1730_Alignments_FINAL.tar.gz   – All 1,730 single-copy ortholog alignments.
- 1730_ML_GENETREEs.treefile – Maximum likelihood gene trees.
- ASTRAL_Tree_AVGdates.tre  – The ASTRAL topology (Figure 1) with average	dates from 10 independent datasets.
- All_Dating_Datasets_DRYAD.tar.gz – The concatenated alignments used for	dating analyses, including the “Concordant Genes” alignment.
- PARSIMONY_1730_Gene_Trees.tre – All 1,730 parsimony gene trees from MPBoot.
- Supp_fig4A_F_s6_b1_v2_1.slim-SLiM3 recipe for Supp. Fig 4A simulation
- Supp_fig4B_F_s6_b1_v2_highmut_1.slim- SLiM3 recipe for Supp. Fig. 4B simulation

*Reviewer #3:

The manuscript submitted by Vanderpool et al. describes the generation of three novel primate genome sequences, the mining of orthologous protein coding genes across 23 published primate genomes and several outgroup genomes, and an in-depth phylogenomic analysis. The authors estimate the phylogeny and divergence times of primates and compare their results to those of previous studies.  The methods and analyses are, for the most part, sound. The results confirm relationships identified in many previous studies on primate phylogeny. The real novelty in this manuscript is the detailed analysis of genealogical discordance and evidence for ILS and ancient hybridization within several lineages of primates. This section of the manuscript is quite strong and I found the analyses and interpretations to be well-reasoned and sound, and provide important insights into this aspect of early primate evolutionary history. I believe that the findings are very interesting and publishable, but
there are a number of aspects of the analysis that could be improved, which I believe will lend greater confidence to their conclusions.*

We thank the reviewer for their encouraging comments. *1.	The new reference genomes are fairly phylogenetically restricted. It might help the authors message if there is some mention as to why the addition of these particular taxa are important or strategic from the standpoint of phylogenetic resolution and hybridization.*

The reviewer makes a good point. We have now added text explaining how these new genomes help us to better detect introgression in three sub-clades of catarrhines (Line 114):

“The three species sequenced here are all Old World Monkeys, and each is closely related to an already-sequenced species. This sampling scheme provides us increased power to detect introgression among each of the sub-clades containing these species.”

*2.	On page 5, it would be helpful to mention the type of sequencing platforms used for each genome.*

We have amended the text to read (Line 120):

“All species were sequenced using standard methods according to Illumina Hi-seq protocols. Additional long-read sequencing was performed using Pacific Biosciences technology for *Macaca nemestrina*.”

*3.	Given the availability of whole genome sequences from so many primates, I was disappointed that the phylogenetic analysis was restricted to only 1,730 single-copy genes, and did not incorporate non-coding sequence alignments. In many cases they could have better ruled out gene tree reconstruction error if the phylogenetic information content of each locus was increased. For example, primate monophyly is supported for only 55% of the gene trees, which in most cases is probably due to lack of phylogenetically informative sites and probable rooting errors. Does gene tree discordance from the assumed species tree correlated with cds alignment length?*

*Also, it would have allowed for an assessment of discordance across a more significant majority of the genome, rather than a small proportion of coding sites. In my opinion, the quality of this report would be markedly improved if the scope of the analysis was expanded beyond protein coding loci to include intronic sequences and the alignments based on genomic sequences rather than inferred isoforms, which are prone to annotation errors. Also, I feel that the authors have missed an opportunity to catalog rare genomic changes (insertion and deletion events) such as retroelement insertions. Because these events occur only once at a single location they are not prone to within locus recombination, and can be analyzed with D statistics. The addition of these types of analyses, particularly with regard to the basal relationships within Cebidae and Colobinae, would strengthen the results.*

We were able to align the majority of protein-coding genes using in-frame codon alignments, but some of these were still quite messy. Site and gene concordance factors are sensitive to poor alignment, so we took great pains to generate the most accurate alignments possible (see response below regarding filtering and alignment steps). Including intronic sequences and other less-conserved regions of the genome would greatly increase alignment error given that we are considering taxa that are >60 million years diverged.

The point regarding gene tree error and cds length is a good one. We investigated this issue by re-calculating concordance factors using the 200 longest alignments in our data set. We have added the following description of the results to the new section of the manuscript, “Sources of Gene Tree Discordance” (Line 354):

“In order to determine the degree to which short alignments or genes with low phylogenetic signal contribute to inaccurate gene trees, we recalculated gCFs and sCFs using the genes with the 200 longest alignments in our dataset (lengths ranging from 1,640 bp to 6,676 bp). The resulting gCFs for the branch leading to the Primate common ancestor increases from 45% to 66% while the sCFs remain unchanged (Figure S1). For the branch placing Dermoptera sister to Primates, using trees estimated from the 200 longest alignments resulted in a modest increase in gCFs from 37% to 45%. Overall the gCFs for the 200 longest genes were higher for all branches in the tree, with the average gCF increasing from 65.18% to 79.74%. The consistent increase in gCF but not sCF when using longer genes points to errors in gene tree inference as a small, but significant factor in our dataset.”

Regarding rare genomic changes, these points are addressed extensively in our response to reviewer #2 above. In summary, such analyses are very hard to do well, but we have endeavored to show here that they do support significant discordance in the two sets of relationships we were able to examine.

*4.	Some important details for the 1,730 loci are missing, such as the chromosome distribution of the loci, whether any are tightly linked, and the breakdown between autosomes and sex chromosomes. Perhaps add a table with this information.*

We have added a new Table S3 to the supplementary material. This table includes the protein name, human chromosome number, and coordinates for each of the human single-copy orthologs.

*5.	I did not see any methods or details surrounding filtration of dubious protein coding sequence alignment regions, given that the gene set is based on the longest isoform from each species, and the ends of isoforms are often poorly annotated. This step is fairly important for the analysis of deeper evolutionary divergences, where alignment anomalies are commonplace and methods have been described to deal with these issues (see Liu et al. PNAS [2017], Mason et al. Sci Adv. [2016], Springer & Gatesy, Systematics and Biodiv. [2018]). Some reporting of these details would be important to have confidence in the assessment of gene and sitewise discordance estimates, and that these values aren't unnecessarily inflated due to poor alignment quality and exon, isoform paralogy. For example, on pgs. 9-10 (lines 199-201) the authors correctly point out the various technical problems (including poorly aligned sequences, etc.) that can result in gene tree error, but they don't provide
any in-depth analysis of their own data set in this regard to determine how often gene tree error can be attributed to these different factors.*

We performed stringent alignment filtering (after extensive testing of various alignment methods), but possibly failed to describe these steps fully in the original submission. We have now added to the text to further describe our filtering steps (Line 687):

“Coding sequences (CDS) for each single-copy orthogroup were aligned, cleaned, and trimmed via a multi-step process: First, sequences in each orthogroup were aligned by codon using GUIDANCE2 [128] in conjunction with MAFFT v7.407 [129] with 60 bootstrap replicates. GUIDANCE2 uses multiple bootstrapped alignments to generate quality scores for each column in the final alignment as well as for each taxon sequence in each alignment. Sequence residues in the resulting MAFFT alignment with GUIDANCE scores < 0.93 were converted to gaps and sites with > 50% gaps were removed using Trimal v1.4.rev22 [130]. Alignments shorter than 200 bp (full dataset) or 300 bp (four-taxon tests for introgression), and alignments that were invariant or contained no parsimony informative characters, were removed from further analyses. Alignments with high numbers of discordant sites were further inspected for errors and removed from the analysis when warranted. This resulted in 1,730 loci for the full analysis (see Table S8 for gene counts used in four-taxon tests).”

We also make all of our alignments available via Dryad, so that researchers can independently assess their quality.

*6.	The use of a single outgroup (and most notably the mouse, which is extremely accelerated in terms of nucleotide substitution rate compared to all other placental mammals) is potentially problematic and may have led to long branch attraction and other artifacts that would increase the amount of discordance pertaining to the relationships between primates, colugos and treeshrews. The authors should add at least one lagomorph; the rabbit and pika genomes are of similar quality and annotation as the primate genomes. Also, in mentioning previous literature, the authors might note that studies that used rare genomic changes (indels and retroelement insertions) produced statistically significant results that are not prone to the same bootstrap issues raised by the authors on lines 171-174.*

This is a good point, and we have therefore added an analysis of a random set of 150 single-copy genes with pika included as an outgroup (reported in the new section on “Sources of Gene Tree Discordance”). Using these 150 gene trees we get the same topology as in Figure 1. While concordance factors are generally unchanged in this set, gCFs for the branch uniting Dermoptera+Primates are actually lower (27.2% vs. 39.5%) with the pika outgroup included (Supplementary Figure S2). The new text reports these results (on line 373):

“Using a single outgroup (mouse), could potentially lead to biases such as long-branch attraction near the base of the tree. To ameliorate these concerns, we performed an additional analysis using 150 randomly chosen single-copy orthologs, with pika (*Ochotona princeps*) included as a second outgroup. As in the full dataset, maximum likelihood and parsimony were both applied to a concatenated dataset, and gene trees were also inferred via both ML and parsimony. Parsimony analysis of the concatenated alignment resulted in the same topology as in Figure 1, while a maximum likelihood analysis produced the same topology as the full ML-CONCAT tree from 1,730 loci, preferring a symmetric tree for the NWM clade. To assess the effect of including an additional outgroup on concordance factors, we calculated gCFs and sCFs using the 150 single-copy orthologs both with (Figure S2A) and without (Figure S2B) pika (using ML gene trees). In contrast to expectations about any error introduced by long-branch attraction, we observe slightly lower gCFs near the base of the tree when pika is included (Figure S2). Site concordance factors are not affected by the inclusion of pika. These analyses indicate that including additional outgroups when analyzing the full dataset is unlikely to reduce concordance factors or to change inferences of the species tree.”

***Minor point, the word "probability" is missing after "posterior" on line 172.*

“probability” was added to the revised manuscript.

*7.	Claiming parsimony (pg. 13) would not produce a biased topology with concatenation methods seems a bit of an overstatement. Parsimony methods are more prone to long branch attraction artifacts than ML methods. The authors should consider qualifying their comment here.*

We recognize these biases, and have therefore changed the text to read (Line 307):

“To investigate these relationships further, we carried out additional analyses. The trees produced from concatenated alignments can be biased in situations with high ILS when maximum likelihood is used for inference, but this bias does not affect parsimony methods [21,72].”

*8.	Line 391, "then re-aligned orthologs present in a single copy in each taxon"…please clarify why this was done.*

To clarify, we have changed the text to read (Line 525):

“To further verify these instances of potential introgression, for each of these seven branches we increased the number of gene trees used, as well as the alignment length for each locus, by subsampling a smaller set of taxa. We randomly chose four taxa for each internal branch tested that also had this branch as an internal branch, and then aligned all orthologs present in a single copy in each taxon.”

__________________________________________________
