# Supplementary material for: Primate phylogenomics uncovers multiple rapid radiations and ancient interspecific introgression
Source: PLoS Biol. 2020 Dec 3;18(12):e3000954. doi: 10.1371/journal.pbio.3000954 (PMC7738166; doi:10.1371/journal.pbio.3000954)
Supplement: Attachment — Submitted filename: Response_to_Reviewers_2.docx [file pbio.3000954.s016.docx]

REVIEWERS' COMMENTS:


Reviewer #2:

This is a re-review by reviewer #2, so I will mainly comment on issues that I brought up in the first round.  Generally, the authors responded to nearly all of my queries with thoughtful responses and edits to their paper, and they included a more than required response to my query about negative selection by completely redoing a published simulation study that they found lacking.  My only two remaining requests have to do with publication of alignments for both genes and transposon insertions. 

The authors note that they have posted their trimmed DNA sequence alignments, and they have clarified how this trimming was done.  However, for other researchers to exactly follow what was originally aligned and then tossed from each alignment is critical for interpretation of their results and conclusions.  This is because many of their conclusions have to do with recombination and possible gene flow.  These inferences derive from conflicts among loci and within loci that are caused by these processes.  So, in order to track what is what in their overall analysis, the precise homology relationships of sequences should be presented both in the original alignments of what the authors thought are homologous regions of each species' genome (to see whether initial annotations of genes are justified) and also in the final alignments that were used in analyses (which the authors have posted already).

We have uploaded the unaligned sequences (All_1735_UNALIGNED_Seqs.tar.gz) to Data Dryad so that decisions regarding homology in the aligned sequences can be made independently by other researchers.

The following files are now available in the Dryad respository (Reviewers link): https://datadryad.org/stash/share/bcYrWxqVDBzeFwMnTfBYgp3k4x0gUlMHby6zJGRcylg

- 1730_ALIGNMENT_CONCAT.paup.nex – Concatenated alignment with PAUP	block of commands used to generate the parsimony concatenated tree.
- 1730_Alignments_FINAL.tar.gz   – All 1,730 single-copy ortholog alignments.
- 1730_ML_GENETREEs.treefile – Maximum likelihood gene trees.
- ASTRAL_Tree_AVGdates.tre  – The ASTRAL topology (Figure 1) with average	dates from 10 independent datasets.
- All_Dating_Datasets_DRYAD.tar.gz – The concatenated alignments used for	dating analyses, including the “Concordant Genes” alignment.
- PARSIMONY_1730_Gene_Trees.tre – All 1,730 parsimony gene trees from MPBoot.
- Supp_fig4A_F_s6_b1_v2_1.slim-SLiM3 recipe for Supp. Fig 4A simulation
- Supp_fig4B_F_s6_b1_v2_highmut_1.slim- SLiM3 recipe for Supp. Fig. 4B simulation
- All_1735_UNALIGNED_Seqs.tar.gz: Unaligned gene sequences.
- plot_DATING.R: R script used for plotting Fig 3.

Second, I think it would be important to post a subset of the alignments for transposon insertions for the position of the tarsier relative to bushbaby and human.  This would be an important contribution, because as far as I know, no shared derived transposons have been documented for the human+bushbaby clade in the literature, but the authors have discovered possibly as many as 341.  Hartig et al. (2013) instead found 104 transposon insertions that cleanly support a human+tarsier clade with no conflicts at this node.  To be blunt, I do not really believe the new result that support for this latter clade (435 transposons) is countered by extreme conflict (341 transposons), because the authors have not looked at the transposon insertion site alignments in detail to assess the quality of the alignments as done in Hartig et al.  Although, I do not expect the authors to go through all of these 341 conflicting transposon alignments as this is not the primary focus of the paper,
they should check a subset of these to be sure that at least a good number of these are convincing in terms of alignment ends, insertion points, homology of the transposon insert, etc. which can only be done currently, I think, by looking at these by eye.  Ideally, it would be great if they could document 10-20 'clean'and convincing  transposon insertions for the human+bushbaby clade and present these alignments in a supplementary file that would document these to skeptics (like me...).

We performed manual inspection of over 100 transposon insertions from our original analysis. As perhaps suspected by the reviewer, this manual inspection was an important step in removing bad alignments. Nevertheless, we were easily able to identify 17 high-quality Alu insertions shared discordantly between human+bushbaby.

As a way to put these in context, we also examined the alignments from concordant Alu insertions reported by Hartig et al. (2013). Our alignments appear at least as dependable as these. Files with alignments of insertions, including four insertions shared discordantly between human+gorilla, are available via FigShare at: <https://figshare.com/account/projects/90941/articles/13105712>.
